# Chronic Hindlimb Ischemia Assessment; Quantitative Evaluation Using Laser Doppler in a Rodent Model of Surgically Induced Peripheral Arterial Occlusion

**DOI:** 10.3390/diagnostics9040139

**Published:** 2019-10-02

**Authors:** Bogdan Hoinoiu, Lucian Petru Jiga, Alexandru Nistor, Vlad Dornean, Sorin Barac, Gratian Miclaus, Mihai Ionac, Teodora Hoinoiu

**Affiliations:** 1 Division of Clinical Practical Skills, Victor Babes University of Medicine and Pharmacy, Timisoara, 300041 Timiș, Romania; bogdan.hoinoiu@gmail.com; 2 Department of Plastic, Aesthetic, Reconstructive and Hand Surgery, Evangelisches Krankenhaus Oldenburg, Medical Campus, University of Oldenburg, 26122 Oldenburg, Germany; ljiga2@umft.ro; 3 Division of Microsurgery, Pius Branzeu Center for Laparoscopic Surgery and Microsurgery, Victor Babes University of Medicine and Pharmacy, Timisoara, 300041 Timiș, Romania; alex3@umft.ro (A.N.); vdornean4@umft.ro (V.D.); sbarac5@umft.ro (S.B.);; 4 Neuromed Diagnostic Imaging Centre, Timisoara, 300218 Timiș, Romania; gratianmiclaus@yahoo.com

**Keywords:** experimental model, hindlimb, chronic ischemia, laser Doppler

## Abstract

Therapeutic neoangiogenesis (TNA) holds promise as a treatment for peripheral arterial disease. Nevertheless, proper tools for in vivo pre-clinical investigation of different TNA approaches and their effects are still lacking. Here we describe a chronic ischemic hindlimb model in rats using laser Doppler quantitative evaluation of tissue perfusion. Male Wistar rats (*n* = 20), aged between 6–8 months, with an average weight of 287 ± 26.74 g, were used. Animals were divided into two experimental groups: group A (*n* = 17; hindlimb chronic ischemia model) and group B (*n* = 3; control). Hindlimb ischemia was induced by concomitant ligation of the right femoral and popliteal artery. Evaluation of tissue perfusion was quantified in perfusion units (PU) on a scale from 0 to 500 (500 PU = maximal detectable perfusion) by laser Doppler analysis at day 0, day 15 and day 30 after induction of ischemia. Induction of chronic ischemia in the rat hindlimb by concomitant ligation of the femoral and popliteal artery can be readily obtained but requires basic microsurgical skills. Laser Doppler analysis has shown unaltered ischemia levels throughout the study (129,17 PU ± 3.13 day 0 vs. 130,33 PU day 30 ± 3,27, *p* = not significant (n.s.)). We demonstrate a simple and reproducible model of chronic hindlimb ischemia in rats, with stable tissue perfusion levels that are accurately quantified using laser Doppler technology. Hence, this model can represent a valid tool for further studies involving therapeutic neoangiogenesis.

## 1. Introduction

Peripheral arterial disease (PAD) represents a major health problem. According to the World Health Organization (WHO), mortality among patients suffering from this disease is over 20%. Moreover, it represents the main cause of major amputations in over 38% of patients within three years of diagnosis [1]. These results are due to the lack of effective treatments, especially in critically ischemic limbs which have evolved beyond revascularization. For this class of patients, major amputation remains the only therapeutic option able to improve clinical symptoms and save the patient’s life [2,3,4]. Under these circumstances, new and more effective therapeutic interventions become mandatory. Early detection and characterization of the disease could improve interventional strategies, leading to a better prognosis in PAD patients. One interesting option is postnatal neoangiogenesis induced by pluripotent stem cells [5]. Accumulating evidence shows that stem cells administered in patients with non-operable PAD resulted in the limitation of ischemic ulcers and significantly reduced the number of major amputations [5], suggesting the potential of this therapeutic approach. However, before these therapies can enter into the clinic, there are certain important issues that require validation in preclinical studies (e.g., the mechanism of neo-vessel formation, the persistence of neoangiogenesis etc.) [6,7]. Here, one of the most important issues is the in vivo experimental model used to assess improvement in tissue perfusion in relation with the neoangiogenic approach.

Although several experimental models of chronic limb ischemia have been documented in different animals, uniformity concerning both the level and method of vessel ligation as well as proper long-term tissue ischemia quantification are still lacking [8,9]. Moreover, aggressive transperitoneal vessel ligation, described to induce adequate ischemia in murine species, has been shown to produce self-mutilation, thus affecting numbers of experimental groups and statistical significance.

Computed tomography angiography (CT–angiography) has high spatial and temporal resolution and its key benefit is that it can provide visualization of vessel anatomy and gross morphology, commonly with a vascular contrast agent. However, it lacks the superior soft tissue differentiation [10].

Here we describe an experimental model of rodent chronic hindlimb ischemia using simultaneous two-level infra-inguinal vessel ligation in which quantitative laser Doppler was successfully employed for long-term quantification of tissue perfusion.

## 2. Material and Methods

### 2.1. Animals and Experimental Groups

A total of 20 male Wistar rats, with an average weight of 287 g (range 258–335 g) were used and divided into two groups: group A (17 animals)—for inducing hindlimb ischemia—and group B (3 animals)—negative control (without ischemia). The allocation of the animals into experimental groups was implemented according to a randomized design. The Animal Facility of Victor Babes University of Medicine and Pharmacy, Timisoara, provided and housed all the animals in a temperature-controlled environment. The rats received water and standard laboratory animal chow ad-libitum. A 12 h light–dark cycle was maintained throughout the experimental protocol. All experiments were conducted with the approval of the Ethics Committee of the Victor Babes University of Medicine and Pharmacy, Timisoara No. 1445/8.07.2010.

At the end of the experiments, all animals were euthanized by intrapulmonary injection of 0.5 mL of T61^®^ (Ebutramid, Intervet/Merck Animal Health, Boxmeer, The Netherlands) and tissue samples were collected in order to investigate expression markers of angiogenesis in the tissues.

### 2.2. Induction of Chronic Ischemia

All maneuvers were performed under inhalatory anesthesia with Isoflurane^®^ by mask (induction: 5% and O_2_ 2 L/min; maintenance: 2% and O_2_ 2 L/min) using a vaporizer (Harvard Apparatus^®^, Holliston, MA, USA). Body temperature was maintained continuously throughout surgery using a heat pad (Temperature Controller^®^, CMA-Harvard Apparatus, Holliston, MA, USA). Surgery was performed under clean, but not sterile conditions, using standard microsurgical instruments.

Preoperatively, the fur from the right hindlimb was removed circumferentially using hair-clippers (Favorita^®^, Aesculap, Tuttlingen, Germany). The animals were placed in the ventral position and the hindlimb was immobilized in extension using elastic loops.

**Figure 1:**
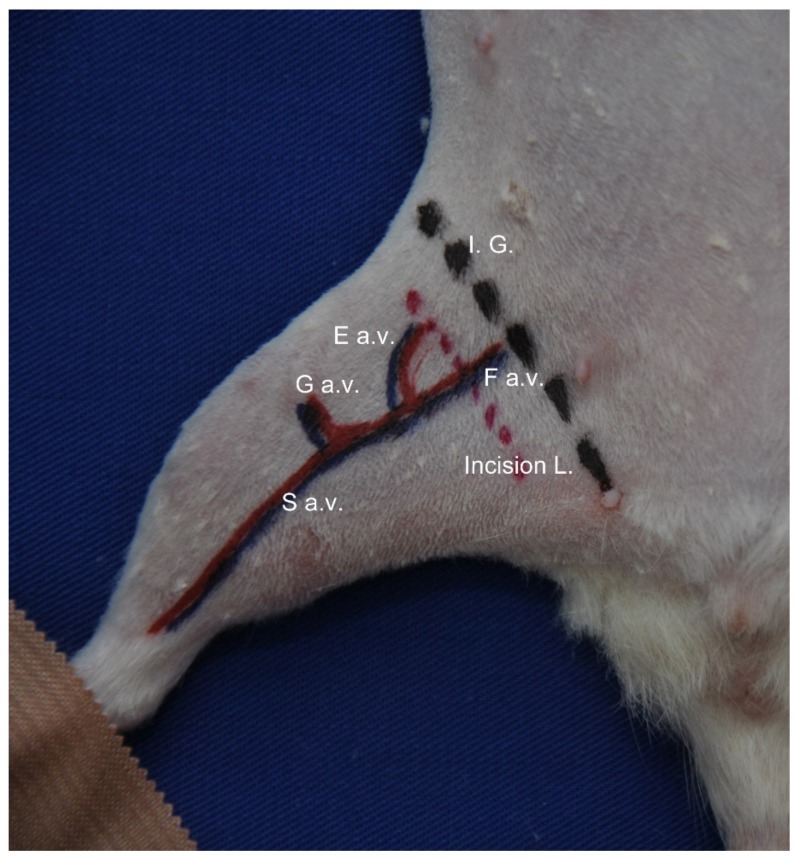
Preoperative marking of the incision area. The anesthetized animal was placed on the surgery board in the ventral position with hindlimbs immobilized in extension. Marking of the right hindlimb was as follows: I. G.—skin projection of the inguinal ligament; Incision L.—the projection of future skin incisions; F a.v.—femoral artery and vein; E a.v.—epigastric artery and vein; G a.v.—geniculate artery and vein; S a.v.—saphenous artery and vein.

The femoral neurovascular pedicle was approached through an incision of about 2 cm, performed at the inguinal fold (Figure 1).

Dissection of the femoral vessels was performed from proximal (the inguinal ligament) to distal (distal to the origin of the saphenous vessels) by circumferential dissection of the femoral and popliteal artery and the proximal third of the saphenous artery. Titanium “S” size clips were used (Vitalitec^®^, Plymouth, MA, USA) to clamp the femoral and popliteal arteries. Finally, the wound was sutured with a single-layer suture using Dafilon 4-0 (BBraun^®^ Melsungen, Germany).

### 2.3. Clinical Evaluation of Ischemia

The retraction reflex to mechanical stimulation of the hindlimb was performed in order to evaluate nociception, according to the semi-quantitative scale described by Schlag et al. [11]. Mechanical stimulation implied compression of finger four and plantar tissue using surgical forceps. Depending on the response to this type of stimulation, a score from 0 (for no nociception—response absent) to 6 (for strong reflex—foot withdrawal) can be recorded. Nociceptive evaluation was performed on day 0, day 15 and day 30. Stimulation was performed four times at intervals of 30 s for each animal to avoid hypersensitivity. Finally, an average score was calculated per experimental group.

### 2.4. Laser Doppler Quantification of Ischemia

For laser Doppler analysis, the MoorLDLS^®^ multichannel laser system (Moor Instruments^®^ Ltd., UK) and the provided software (MoorSOFT for Windows^®^, Moor Instruments Ltd., Axminster, UK) were used, according to manufacturer instructions. Anesthetized animals were placed on the scanning table and the hindlimbs were analyzed using the following settings: 15 cm—measuring distance, 256 scanning lines, 100 ms/line—measuring speed, 400 contrast units. Tissue perfusion was quantified in perfusion units (PU, range 0–500) within pre-defined ROIs (regions of interest), covering the entire analyzed limb. Since all animals in the study were of the same age and average weight, a standard ROI was defined by a polygonal shape encircling the entire hindlimb at approximately 2 mm from its actual cutaneous borders, extending the root of the extremity medially and superiorly as defined by the cutaneous projection of the inguinal ligament. The same laser Doppler (LD) measuring settings and pre-defined ROIs were used for all experiments. 

Laser Doppler analysis was performed preoperatively, on day 0, day 15 and day 30 for the group of animals that underwent surgical vessel ligation at the right hindlimb (group A), as well as for the control group (group B). Results of tissue perfusion quantification (measured in PU) are presented as average per ischemic limb/animal/day 0, day 15 or day 30 ± standard deviation (SD).

### 2.5. Computed Tomography Angiography (CT–Angiography)

After open cannulation of the external jugular vein using a dedicated 3 Fr catheter (Harvard Apparatus^®^, Holliston, MA, USA), the dorsal injection port was tunneled at the level of the interscapulovertebral area.

The anesthetized animals were placed in the dorsal position on the examination table and the central venous catheter was connected to the automatic injection of contrast dye. The injection (5 mL Ultravist 370^®^ (Bayer Healthcare Pharmaceuticals Berlin, Germany) + 4 mL NaCl 0.9%, rate of 0.8 mL/s) was administered while scanning. Image acquisition was performed with 64 sections × 0.6 mm, and the images were post-processed using the volume rendering technique (VRT) three-dimensional (3D) interface. Acquisition and post-processing of images was performed using a Somatom Sensation 64^®^ type computer tomography (Siemens AG, Munich, Germany) and Siemens syngo^®^ software (image acquisition), respectively. Multimodality Workplace^®^ was used for image post-processing and interpretation of results.

### 2.6. Statistical Analysis

Values are presented as the mean value calculated per group, at day 0/15/30 ± SD. All experiments were performed at least three times. Statistical significance was calculated using SPSS 8.0 (SPSS Inc., Chicago, IL, USA) with Log Rank and Mann–Whitney tests. Differences were considered statistically significant at *p* < 0.05.

## 3. Results

### 3.1. Postoperative Survival and Operating Time

During the follow up period, the investigators confirmed the health state of the animals and survival twice a day. Three animals died in the postoperative period (day 11, day 12 and day 23) and one was excluded due to the self-mutilation phenomenon of the ischemic limb (day 18). The average operating time for inducing ischemia was 38.4 min ± 5.1, *p* < 0.001. 

**Figure 2:**
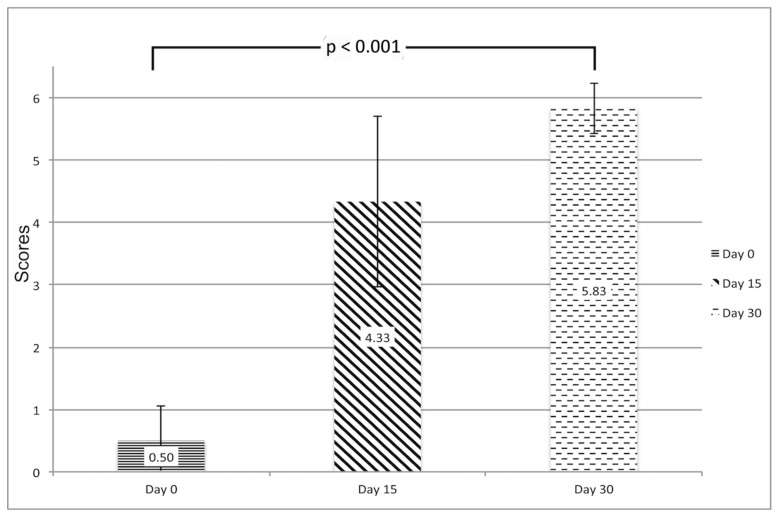
Semiquantitative assessment of the degree of ischemia in response to painful stimuli. The graphic represents the degree of perception of painful stimuli at the hind ischemic limb on day 0, day 15 and day 30 (group A). A recovery of nociceptive sensitivity has been observed (0.5 ± 0.55 on day 0 vs. 5.83 ± 0.41 on day 30). Data shown as mean ± standard deviation (SD).

According to the semi-quantitative analysis of the degree of ischemia in response to painful stimuli, on day 0 all animals in group A had major sensitive palsy of the ischemic hindlimb, but 83% had regained sensitivity on day 30, with an average score of 5.83 ± 0.41 on day 30 compared with an average score of 0.5 ± 0.55 on day 0 (*p* < 0.001). (Figure 2).

### 3.2. Laser Doppler (LD) Quantification of Ischemia

The induction of chronic ischemia was examined by analyzing postoperative tissue perfusion in the segment of interest (right hindlimb) within pre-defined ROIs using the laser Doppler technique on day 0, day 15 and day 30.

**Figure 3:**
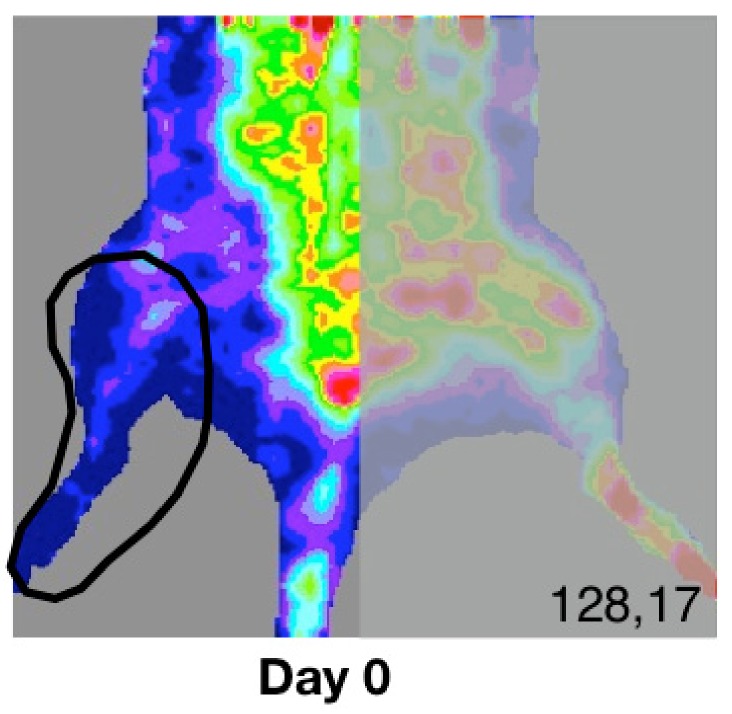

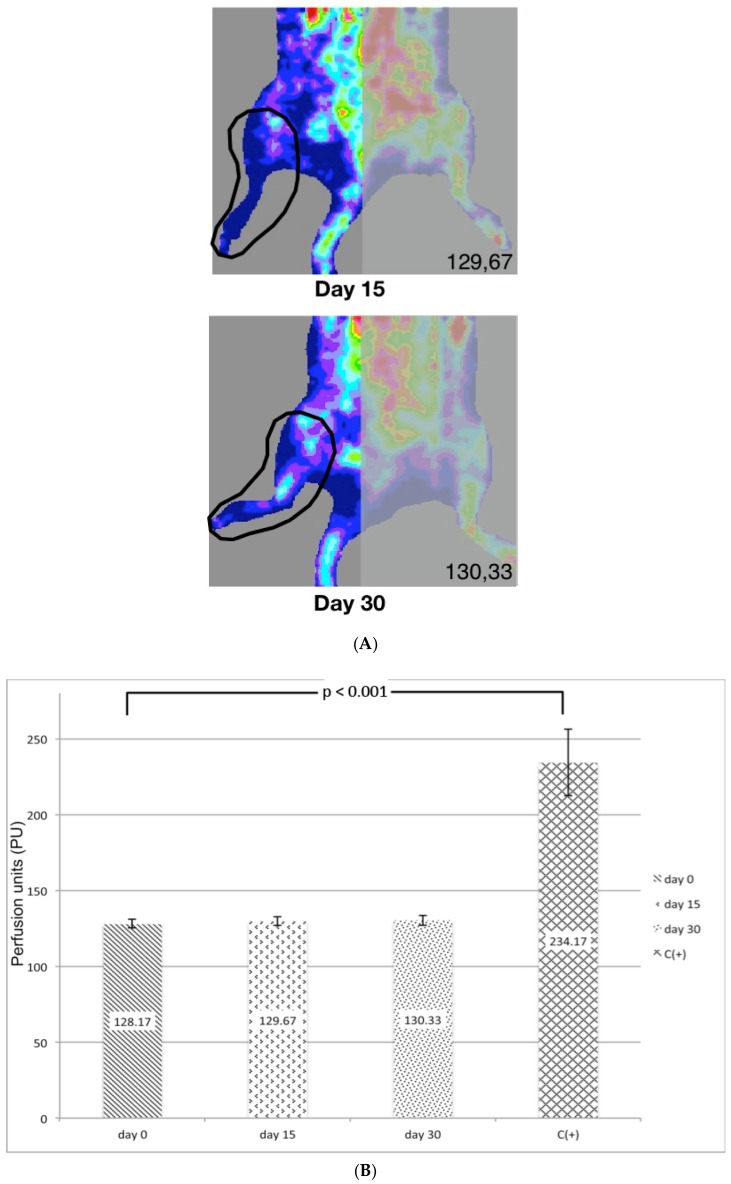
Animals in group A underwent the surgical procedure on day 0. Laser Doppler analysis quantified the degree of ischemia on day 0, day 15 and day 30. (**A**) Thermographic map and region of interest (ROI) comprising the tissue area analyzed on day 0, day 15 and day 30. The picture presents the same animal throughout the experiment. (**B**) Mean perfusion units for group A on day 0/15/30 ± SD. Day 0 indicates values comparable to day 30. (128.17 PU ± 3.13 vs. 130.33 PU ± 3.27 recorded on day 30, *p* = n.s.). C(+) = positive control.

The results show constant low tissue perfusion in the ischemic hindlimb throughout the study. According to the thermographic analysis (Figure 3A,B), on day 0 there is a value of 129.17 PU ± 3.13 vs. 130.33 PU ± 3.27 recorded on day 30 (*p* = not significant (n.s.)).

### 3.3. CT–Angiography

**Figure 4:**
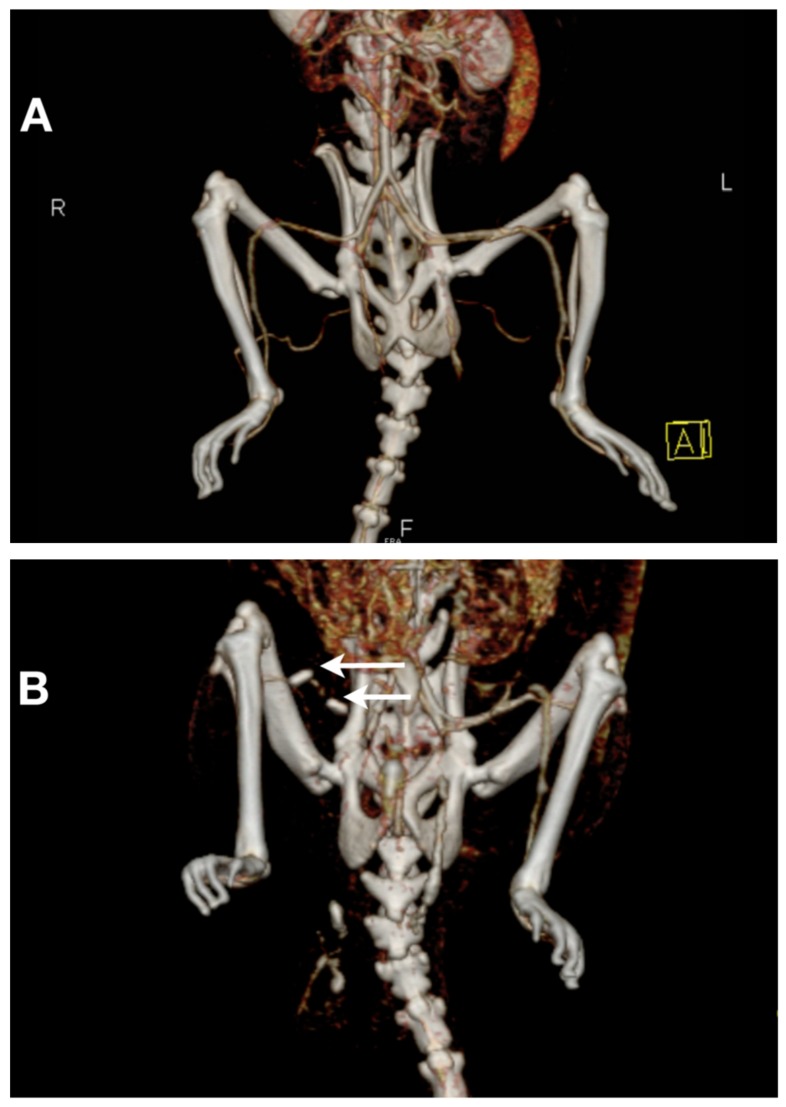
Computed tomography (CT)–angiography demonstrated the absence of collateralization at day 30. (**A**) Volume rendering technique (VRT) images of the hindlimb of a rat from the control group with normal vascularization. (**B**) Image of an animal from group A showing the titanium clips (indicated by arrows) on right common femoral and popliteal artery and also the absence of distal perfusion and collateralization.

CT–angiography was performed for all animals in group A on day 0 and day 30. On day 30, VRT images indicate complete mechanical obstruction of the common femoral and popliteal artery and the absence of collateralization (Figure 4A,B). These results are consistent with severe persistent ischemia diagnosed by laser Doppler quantitative assessment.

## 4. Discussion

There are several experimental models of peripheral arterial occlusion described in the literature, which vary depending on the method used to induce ischemia or the blood flow evaluation method [12]. Main differences are related to: vascular segment (e.g., femoral and/or iliac), the method used (e.g., artery ligature or excision), and blood flow which, in rodents, can normalize at different time intervals from several minutes to five days after iliac artery ligation [12,13].

Hindlimb blood flow remains unchanged after a simple ligation of the common iliac or femoral [14] artery of rats [15]. Siefert et al. have shown that ligation of the common iliac artery and its branches causes a reduction in blood flow, but only for five days [13]. In comparison, Hendricks et al. [8] reported prolonged reduction in blood flow for two weeks after ligation of the common iliac artery in rabbits. Alternately, using pigs in a hindlimb model of single femoral artery ligation, Buschmann et al. have shown reduced blood pressure and blood flow for two weeks [9].

According to the existing literature, only a limited number of stable chronic hindlimb models of ischemia in rodent species have been published. Rodent species develop collateral vessels a short time after induction of ischemia at one (femoral) or more levels (femoral/iliac). The degree of persistence of ischemia is not conclusive because of different types of protocols/values used [16,17]. Using a rat hindlimb ischemia model, Couffinhal et al. had to employ proximal ligation of the femoral artery and distal saphenous artery followed by complete excision of these vessels to prevent further collateralization and achieve stable tissue ischemia [16].

In contrast, the model described in this paper proves that at day 30, the collateralization is absent (according to CT–angiography) and the ischemia remains constant (129.17 PU ± 3.13 on day 0 vs. 130.33 PU ± 3.27 on day 30, *p* = n.s.).

Hellingman et al. assessed the degree of ischemia by interrupting the blood flow at different levels with different techniques of vascular occlusion: single coagulation of the femoral artery, total excision of the femoral artery, and double coagulation of the femoral and iliac arteries. The results indicate that at four weeks after surgery, the femoral artery excision is the most effective method of inducing ischemia; 46% of the animals studied showed similar levels of ischemia on day 0 [18].

Here, we have shown that concomitant ligation at two different levels (femoral and popliteal) determines persistent ischemia at the infra-inguinal level throughout the study in 100% of animals compared to the 46% found by Hellingman et al. [18].

This study reconfirms the results obtained by Hellingman et al., namely that there is no need for ligation in supra-inguinal territory (e.g., the iliac artery) to induce persistent ischemia in rodents.

The experimental model described here is easy to perform in terms of technique, as well as in terms of persistent ischemia, a fact reasoned by the level of tissue perfusion as measured by laser Doppler analysis. The method here is less invasive compared to other described techniques of ischemia induction that involve a more aggressive transabdominal approach of the iliac arteries. This aggressive approach can lead to limb loss due to self-mutilation [19].

A limitation of this study is the limited tissue penetration of the Laser Doppler equipment. The depth of the tissue explored is limited to around 1 mm, which would miss collaterals that may have developed deeper in the thigh and calf muscles outside the reach of this instrument. Other methods allowing deeper penetration such as high frequency magnetic resonance flow imaging could provide a more accurate assessment, albeit with much higher equipment costs. We do believe, however, that because the skin is vascularized mainly from perforating arteries from deep tissue, laser Doppler quantification can be reasonably extrapolated as a measurement of blood perfusion in the entire hindlimb. Hence, we believe that Laser Doppler continues to provide a satisfactory, rapid and affordable assessment of the microcirculation.

An important variable in laser Doppler determination is the skin temperature during scanning; knowing that immediately after the induction of anesthesia, body temperature drops [15], it was therefore necessary to use a heat pad. Removing the fur and also conducting the measurements under the same temperature conditions are important factors to be taken into consideration to assure uniformity of all measurements.

In the experimental model used here, laser Doppler is an efficient and accurate method to quantify post-ischemic tissue perfusion in the rat hindlimb.
